# Research of Malting Procedures for Winter Hard Wheat Varieties—Part I

**DOI:** 10.3390/foods10010052

**Published:** 2020-12-28

**Authors:** Vinko Krstanović, Kristina Habschied, Krešimir Mastanjević

**Affiliations:** Faculty of Food Technology, Josip Juraj Strossmayer University of Osijek, 31000 Osijek, Croatia; vkrstano@ptfos.hr (V.K.); kmastanj@gmail.com (K.M.)

**Keywords:** wheat, standard malting procedure, restrictive malting procedure, intense malting procedure, wheat malt quality

## Abstract

This paper examines the influence of the malting process of red hard wheat varieties (which have many characteristics of soft wheat varieties and represent a transitional form between durum and soft wheat). According to the values of total and soluble proteins and viscosity of wort these wheat varieties belong to the second malting quality group. To establish the individual response of each variety and estimate how the chosen varieties respond in groups to different process conditions, sixteen varieties were selected and malted according to the standard procedure (A), restrictive procedure (B), and intense procedure (C). Starting wheat, indicators of micromalting process success, and finished malts were analyzed. It was found that the restrictive procedure (B) gives poor results for the values of proteolysis performance parameters (soluble N, free amino nitrogen (FAN)) with simultaneous disturbance and values of cytolytic degradation (viscosity and filtration time) and extract yield. At the same time, this procedure lacks a stronger individual response of an individual variety to the process conditions during malting (F/C difference and extract yield). The optimal malting process for the specified assortment would include the modification of processes B and C in a way to alleviate the restrictive conditions in process B, or in a way to reduce the intensity of the decomposition in process C.

## 1. Introduction

Wheat has been known as a raw material for malting for a long time; brewing varieties from the assortment available in certain countries have adapted to different climatic conditions. The main properties of wheat as a malting raw material are given in a review paper by Faltermaier et al. [1]. Wheat varieties suitable for brewing are purposefully selected. The application of prescribed agrotechnical cultivation measures conditions the quality of wheat malt, but the malting quality can vary depending on the season (environmental conditions) and location. For instance, there are several (mostly German) brewing varieties that are not suitable for use in the area of Southeast Europe. The quality requirements of wheat malt from non-brewing wheat has been discussed in many studies [2,3,4,5,6]. Soft varieties are generally recommended as brewing varieties due to their lower protein content [7], but they are not as well represented in the production in European countries. It is important to emphasize that the current classification of wheat (soft, hard; red, white; bred, confectionery, and livestock feeding) can lead to certain ambiguities because it is difficult to classify varieties of differing origin (both in terms of selection and response to different climatic areas) in these groups. This is especially true of the usual European assortment in comparison to, for example, American or Canadian classifications for durum wheat types [8,9]. Namely, European winter wheat varieties have lower protein levels than the spring varieties [10]. An example of this is that most red, hard wheat varieties grown in the Pannonian Plain (the typical continental climate with frequent “forced maturation” phenomena) are formally characterized as “hard” but have many characteristics of soft wheat varieties. These varieties are characterized by a very pale red color, moderate hardness, almost completely transient vitreous grain, and the absence of awn. The majority of these varieties belong to the II qualitative malting group (according to the German professional classification for the use of wheat as a malting raw material characterized by elevated total and soluble proteins and suitable wort viscosity) [11]. First malting quality group completely fits the malting process conditions (values for soluble N and malt viscosity meet the low values required in this group), and within this group individual varieties can be classified according to the recommended values for individual quality indicators. In II malting quality groups, a problem with an increased content of soluble N can be expected, and when malting varieties from this group, specific process solutions during malting should be applied in order to suppress the excessive proteolysis without the adverse effect, or with a minimal negative impact on other quality indicators (e.g., malting with a moderate increase in germination temperatures). The influence of the malting process itself on the quality of the finished malt obtained from soft wheat varieties was investigated in detail by Sacher [12]. However, the malting procedures recommended for these varieties cannot be applied to domestic varieties, as they differ significantly in terms of genetic potential and required agrotechnical cultivation procedures. For the classification of wheat suitable for malting, two basic malting procedures have been developed according to which the examined varieties should clearly show their response in terms of improving or deteriorating the quality of malt in relation to malt obtained by the standard malting process [10,11]. These are processes with rising germination temperatures (so-called restrictive process) and falling germination temperatures (so-called intensive process).

This paper aims to establish how these processes affect the quality indicators of finished malt for the chosen domestic assortment of wheat varieties (transitional type between soft and hard), as well as how their malting properties can be improved by the malting process itself. A study was therefore conducted into the influence of different process conditions on the malt quality of domestic wheat varieties in order to determine the most favorable impact on the quality indicators of the malting process itself. Part I of this research examined the effects of a restrictive malting process denoted as B (increasing germination temperatures with standard grain moisture), as well as an intensive malting process denoted as C (decreasing germination temperatures, lower humidity during germination stage) on the quality of the finished wheat malts, with special reference to indicators that are crucial for classifying varieties into appropriate malting quality groups. The results for the malt quality indicators obtained by the standard malting process according to the Middle European Brewing Analysis Commission (MEBAK) (denoted as A) were considered as reference. Furthermore, the possibility of obtaining the best possible malt quality is carried out by modifying these two methods (moderately intensive, denoted as D and moderately restrictive, denoted as E). The results are presented in Part II of this research.

## 2. Materials and Methods

The sixteen wheat varieties selected for this research are designated as *Triticum aestivum* L. (ssp. *vulgare*), red grain var. *erythrospermum* or var. *lutescens*. The selection was based on the results of 32 previously tested varieties obtained from the Agricultural Institute Osijek and Bc Institute Zagreb. All the selected varieties in this research belong to the red hard wheat breeding lines/cultivars with promising good malting attributes. Preliminary examination established that there are no varieties in the examined assortment that meet the criteria of the 1st malt quality group, but that almost all varieties that showed satisfactory malt performance according to the standard MEBAK micromalting procedure [13] belonged to the 2nd malt quality group. Sixteen cultivars that showed the best values for the indicators such as soluble N (<730 mg/L) and viscosity (<1.65 mPa × s) of wort, were selected and numbered 1–16 (1-Maria; 2-Liberta; 3-Nina; 4-Adriana; 5-Lana; 6-Ema; 7-Lucija; 8-Ana; 9-Srpanjka; 10-Žitarka; 11-Superžitarka; 12-Barbara, 13-Panonka; 14-OS376-99; 15-OS51-98; 16-Contur). Among these were varieties that showed the best malting properties, i.e., gave the highest quality malts in the aforementioned research. Varieties are EU/Croatian winter red durum wheat that have many more properties of soft wheat and are far from the “hard red” USA and Canada type. These are wheat varieties that give a relatively low concentration of soluble N in wort (relative to the total starting N in malt) and excellent values for malt viscosity. Wheat samples were collected as untreated processed grain (1st class grain, with very good germination energy for 3 days (˃95%) and 5 days (˃98), 10 kg from each variety for two seasons 2018/2019). To avoid the influence of microbiological contamination on malt quality, raw material control concerning *Fusarium graminearum* and *Fusarium culmorum* contamination was conducted, according to the MEBAK (Method 2.6.). Grains were packed in paper bags and stored in a dry and cool place for 2 months to overcome the grain dormancy. All analyses were done in duplicate and according to the Analytica-European Brewery Convention (EBC^®^) [14] and the Middle European Brewing Analysis Commission (MEBAK^®^) [15] methods shown in Table 1, except for the total and soluble pentosanes that were determined according to Shogren et al. [16]. The ability to absorb larger amounts of water leads to more intense swelling and breakage of starch granules, which enables easier enzyme degradation; thus, the variety shows higher enzymatic power. The obtained values were significantly >33%, the minimal value a grain should show after soaking. The ability of wheat to adsorb water was determined according to MEBAK method 2.4.4., as the moisture of grain after 48 h, for all three water temperatures (standard micromalting t_a_ = 14.5 °C, and two higher temperatures t_b_ = 16 °C and t_c_ = 18 °C).

Micromalting was carried out in a micro malting plant (Joe White Malting Systems Pty Limited, East Melbourne, Victoria, Australia) using an Automatic Micro Malt Unit according to the scheme shown in Table 2. Procedure A was the standard MEBAK procedure (Method 2.5.3.1) with the correction of air humidity during the dry steeping phase (85%); this is because wheat lacks the thick barley husk and absorbs water more quickly. On the third day of soaking, the grain was transferred to the germination box where the humidity of the grain was adjusted by sparging. The restrictive procedure, B, included increasing germination temperatures; C was the intensive procedure with decreasing germination temperatures. These terms refer to the degree of grain moisture at the beginning of germination and the temperature during the germination phase.

In all malting processes, the grain was subjected to the germination conditions on the third day of steeping. The required grain moisture was adjusted by sparging the grain during germination until it reached the required moisture. In this way, the last day of soaking was also the first day of germination. After the germination, the malt was dried according to the standard MEBAK procedure for light malt. After micromalting and degermination, malt was stored for one month to stabilize the moisture content and enzyme activity. Values of proteolytic, cytolytic, and amylolytic quality indicators of finished malt were used to assess the malt quality of a particular variety [12,17]. Analyses of malt quality indicators were done according to [14,15].

Data analysis: differences between the average values of the raw material, micromalting process indicators, and finished malt quality indicators were analyzed using the analysis of variance (ANOVA) and Fisher’s least significant difference test (LSD), with a statistical significance set at *p* < 0.05. Statistical analysis was carried out using Statistica 13.1. (TIBCO Software Inc., Palo Alto, CA, USA).

## 3. Results

The starting wheat intended for malt production can be, according to quality indicators, described as a soft wheat variety with low protein content [11]. This study examines the impact of the malting process on the transitional form of (hard-soft) red winter wheat, which was found to be classified in the II malting qualitative group by the standard MEBAK micromalting process. For these cultivars, an increased share of total and soluble N in malt was found, as well as a strong correlation between total protein share and NIR (Near InfraRed) grain hardness. The correlation between total pentosanes and NIR hardness was somewhat lower [13], which are not unusual results for hard wheat [18]. Furthermore, it was found that the examined assortment had the so-called transient vitreosity which is characteristic of soft wheat (Table 3). Elevated nitrogen values are expected for this qualitative group [12], but compared to the northern European assortment, the Southeast European assortment is exposed to almost regularly forced maturation phenomenon, resulting in a lower proportion of soluble proteins and soluble pentosanes relative to their total values in grain [19,20]. Forced maturation of grains occurs at high air humidity and high temperatures during the grain filling phase. This phenomenon generally impairs the values of almost all grain quality indicators [21]. The examined assortment showed acceptable values for total pentosanes and the ratio of soluble pentosanes/total pentosanes (Table 3). Wheat grain has lower β-glucan content than barley grain but has more arabinoxylans which increase wort viscosity. According to Narziß [22], the viscosity of wort should range from 1.65 to 1.85 mPa × s. For this study and taking into account the previous studies values ≤ 1.65 mPa × s are considered optimal for wort viscosity. No significant correlation between the total and soluble pentosanes was detected. It is also known that soluble pentosanes influence the wort viscosity and membrane filterability (*r* = 0.98) [23,24,25]. The assumed malting process which would be successful in obtaining a better-quality wheat malt from the selected varieties should be a restrictive malting process that would suppress the excessive proteolysis in the grain [11,12]. This process is expected to reduce the concentration of soluble N with acceptable wort viscosity. On the other hand, the intensive malting process should result in a deeper cytolytic degradation, which is consequently accompanied by an increase of viscosity and soluble N value. When comparing the results of the mean values for the quality indicators of the malting process for all tested varieties, a very strong influence of the malting process can be noticed on their values (Table 4).

If we look at the grain moisture after 48 h of steeping (as a measure of grain swelling capacity), we see that procedure B gives the lowest values; a fairly uniform and weak individual response of varieties can be observed. Procedure C gives a stretched-out range of values because of the stronger individual response of each variety and, in total, higher mean values for the whole assortment. This is not surprising because a more intensive malting process leads to a stronger activation of the individual cytolytic and general enzymatic potential of each variety. Procedure A was very similar to procedure B in its mean values, but with a more significant individual response for each variety. In general, considering the character of each procedure, the expected results for the swelling capacity were obtained, as well as for the relationship between the mean values for each procedure. When observing the losses during malting as well as their structure, it is clear that the lowest total losses occurred in process B while process C has expectably significantly higher losses. In procedure B the ratio of germination losses: respiration losses is about 80%:20%, in procedure A 74%:24% and in procedure C 60%:40%. This usually means that higher amounts of energy have been employed for germination (more sugars have been spent) for germination than it is desirable. In practice this means less fermentable sugars for yeast during fermentation. When observing the individual response of a variety, differences between individual varieties according to a certain malting procedure are observed (Table 5, Table 6 and Table 7). When compared to standard procedure A, the results indicate that there were clear varietal responses: grain moisture after 48 h was significantly reduced by procedure B (only varieties 3, 8, and 12 had an approximate value between procedures). Looking at procedure C, slightly higher results were obtained with much more cultivars who had approximately the same value as in procedure A (1, 5, 6, 7, and 14). All cultivars displayed lower and significantly lower respiration losses (lower for 3, 7, 8, 9, and 16; significantly lower for 1, 3, 4, 5, 6, 10, 11, 12, 13, 14, and 15). In procedure C, respiration losses compared to procedure A were higher (4, 7, 12, 13, 15, and 16), significantly higher (1, 2, 3, 6, 8, 9), equal (5), and lower for a variety 10. Germination losses were significantly lower in procedure B, except in variety 2 where they were equal to A. In process C, very different results were obtained (higher for 1 and 6; significantly higher for 2; equal for 3, 9, 12, 13 and 15; lower for 4, 7, 8, 10, 11 and 14; and significantly lower for variety 16).

Losses in procedure C are significantly higher compared to the other two procedures and in general, they are not acceptable from the malting point of view. Total losses in procedure B were significantly lower for all varieties compared to the standard procedure, while the results for procedure C indicated different results for individual varieties. This implies that the intensive procedure provokes a strong individual response from varieties that expectedly exhibit varietal characteristics during the intensification of malting process conditions.

Reviewing the quality of finished wheat malts obtained by these procedures, the individual impact of the applied procedures on each variety as well as the impact on the mean values for the whole assortment were considered. A similar group response of varieties (mean values of quality indicators) according to a certain malting procedure was expected. Namely, in the examined assortment there are no varieties that would fit the criteria for I qualitative group or which could be classified in, e.g., III qualitative group (characterized by a profound cytolytic degradation and increased wort viscosity), which would significantly improve the quality of the obtained malts during the restrictive malting process (B). To compare the malting procedures, we will first consider the ratio of mean values for the quality parameters of the obtained malts, as the classification into qualitative groups is performed (total and soluble N, the viscosity of wort). Wheat malt should not contain more than 2% of total N (<12%) in the grain [22], but higher values were obtained in all malting processes applied in this research. The preferred share of soluble N varies from author to author (650–780 mg/100, [17]; 849 to 1246 mg/L and FAN (free amino nitrogen) from 135 to 185 mg/L [26]. Faltermaier et al. [23] reported that the common content of soluble N in a typical pale malt should be between 600 and 800 mg/100 g, which is approximately 660–895 mg/L. FAN values should range between 100 to 140 mg/100 g, which equals approximately 110 to 160 mg/L. It was expected that malting process B would result in lower values for soluble N in the wort without significantly impairing other quality indicators, primarily the viscosity of the wort.

The results of the analysis of the finished malts are given in Table 8, Table 9, Table 10 and Table 11. Recommended values in Table 8 are from different literature references [12,15,27,28].

The total soluble N share in the grain is greatly influenced by environmental factors. For winter wheat varieties, Psota et al. [2] determined the percentage of different factors that affect the total soluble N share in grains: factor “year” affects it by 61.7%, factor “location” by 14.4%, and factor “variety” by 11.3%. As this was a one-year study at the same location and the varieties were classified in the second malting qualitative group in the previous study, the impact on total N and soluble N depends primarily on the malting process. When looking at the values for other indicators of proteolysis performance (Kolbach Index, FAN, wort color) it is noticeable that process B overly suppresses proteolysis. According to [17], the value of the Kolbach index in the winter wheat varieties was affected by “year” by 52.3% and by “location” by 17.2%. The values obtained in this research were within the aforementioned values for all malting processes.

Amino acids are an important yeast-growth factor while polypeptides affect foam stability and quality and contribute to the fullness of beer [28]. Considering that the FAN concentration is high enough, yeast can obtain more nutritive compounds resulting in faster fermentation and higher alcohol production [29]. However, undesirable flavors can be the general outcome of high FAN content [5]. Winter wheat varieties have an equally lower share of FAN when compared to spring varieties [2,3]. From the obtained values for all malting processes, it can be noticed that the tested assortment showed lower values than the recommended ones. Although slightly higher values were obtained by process C, the malting process did not significantly influence these factors. The most important indicator of amylolysis is the extract yield. The average obtained values for extract yield for winter wheat are slightly higher than in winter barley (82.3–85.4%) [2,3]. This corresponds to the range of 83–85% reported in the literature [5,28]. The highest values for extract were obtained in the process C, but this is not applicable since many other indicators did not show satisfactory values. Process A also resulted in acceptable values for extract yield. The degradation of starch is characterized by final attenuation and the average values for this parameter for winter wheat varieties fall between 78.6% and 81.4% [2,3,22]. For the evaluation of malting quality of wheat varieties investigated in this study, values of final attenuation of <80% are considered unacceptably low while values >83% are optimal [2]. It is interesting that in all malting processes for almost all varieties, the same or higher values were obtained. In spring and winter wheat varieties, diastatic power achieved the average values from 250 to 410 WK [2,3,28]. The obtained values in all malting processes are expected because the southern European assortment has, as a rule, lower enzymatic strength compared to the northern European.

If we add the worst values for cytolytic parameters (viscosity and filtration time), and the lowest values for the yield of the extract (Table 8), it can be stated that a strong restrictive malting process (B) is not acceptable for wheat belonging to II malt quality group; it should be modified in such a way as to deepen the decomposition of the grain. In this way, a stronger individual response of each variety would be encouraged, as observed in process C. This is particularly important to assess the malting potential of each variety as accurately as possible. Intensive process C is expected to lead to a greater stretching of the results as a result of stronger activation of individual enzyme potential without excessive increase in viscosity. However, given the total dry matter losses expressed as a comparison of 1000 grain weight between these three processes and values for extract yield, the highest value for total N in malt this process proved to be less acceptable than standard procedure A and also requires correction in terms of reducing the depth of degradation with satisfactory values for the cytolytic and proteolytic complex.

To assess the individual response of a particular variety to changes in process conditions during malting (Table 9, Table 10 and Table 11), the improvement of the quality of malt obtained from a particular variety was compared with malt obtained by standard procedure A. In doing so, there was a tendency for variety to skip from qualitative group II in quality group I (total soluble N < 770 mg/L and viscosity <1.65 mPs × s). When considering proteolytic parameters (soluble N, FAN, wort color, pH) for the examined assortment and applied malting procedures, it was found that process B in general and in all cultivars results in an increase of soluble N in malt. However, many cultivars had approximate value as in process A (1, 7, 11, 12, 14, 16). This increase in the proportion of total N in malt can be explained by poor proteolysis, which consequently leads to poor protein degradation into low molecular weight compounds that can be metabolically utilized for germination and respiration. The results for soluble N, which was significantly lower in almost the entire assortment, confirmed this; only varieties 1, 2, and 3 had an approximate value as in standard procedure A. A significant number of varieties resulted in an increased share of total N in malt and increased viscosity in wort followed by significantly longer filtration time. This is common in varieties that further research showed as having the best malting characteristics when subjected to moderately restrictive malting process (8, 9, 10, 11, 13, 14, 15, and 16) (data shown in Part II). As for FAN, it can be noticed that process B did not provide sufficient proteolysis especially for the formation of amino acid N, which ultimately reflects as slightly elevated wort pH when compared to process A. Furthermore, some varieties were found to have shorter filtration times in addition to the increased viscosity (varieties 6, 7, 8, and 9). Process B generally resulted in higher vitreosity than process A (varieties 2, 8, 10, 11, and 12). No significant correlation was found between the limit of attenuation and other indicators of the quality of the finished malt, which agrees with the previous research [13]. In the intensive process, C, a higher share of total N in malt was observed and in a large number of varieties, when compared to process A (Table 10), which is similar to the trend in process B has another reason. In process B the insufficient proteolysis led to less low molecular weight nitrogen compounds, affecting the metabolism during germination. In process C, a profound cytolytic and amylolytic degradation led to a change in the starch: protein ratio in favor of protein. Namely, these two compounds are formally correlated in grains, which roughly means that they complement each other up to 100. The excessive starch consumption (which can be seen from the total losses during malting (Table 4 and Table 7), extract content and extract difference reported in Table 11) results in a formal increase in the share of total N in malt. Soluble N was generally higher in almost all cultivars while the results for FAN varied from cultivar to cultivar but did not differ significantly from the values obtained by method A. In almost all cultivars a significant reduction of filtration time and viscosity of wort occurred. The pH of the wort is intertwined with a number of parameters, so it is difficult to interpret the results. However, its reduction is presumably related to protein degradation products (primarily low molecular weight and amino N). A similar explanation can be applied for the color of the wort.

## 4. Conclusions

When malting hard red wheat characterized as II qualitative malting group, the intensive restrictive procedure (B) gives poor results for proteolytic degradation (soluble N, FAN) and disturbs the values of cytolytic degradation (viscosity and filtration time, F/C difference) and extract yield. Intensive process C provides a stronger individual response of each variety, as well as lower values for soluble N and malt viscosity, but does not significantly improve the values of malt quality parameters when compared to standard procedure A. The optimal malting process for the tested assortment should include a modification of procedures B and C in a way to alleviate the restrictive conditions in process B and reduce the intensity of the grain degradation in process C. This was done in the second part of our research.

## Figures and Tables

**Table 1:** Used the Middle European Brewing Analysis Commission (MEBAK) and European Brewery Convention (EBC) methods for the analysis of wheat and malt.

		Method	
	Unit	MEBAK^®^	EBC^®^
Micromalting		2.5.3.1	
1000 grain weight	g d.wt.		3.4/4.4
Moisture	%		3.2/4.2
Fine extract content	% d. wt.	4.1.4.2.2.	
Extract difference	%	4.1.4.2.10	
Saccharification time	min	4.1.4.2.4.	
Filtration time (min)	min	4.1.4.2.5.	
Total N	% d. wt.	4.1.4.5.1.1.	
Soluble N	mg/L		4.9.1
Kolbach index	%		
Hartong number VZ 45 °C	%	4.1.4.11.	
Final attenuation of wort	%		4.11
Wort colour	EBC	4.1.4.2.8.2.	
Viscosity	mPas. 8.6%e	4.1.4.4.2.	
Diastatic power	°WK	4.1.4.6.	
Vitreosity	%	4.1.3.5.1	
FAN ^1^	mg/100g malt dry m.		4.10
pH	-	4.1.4.2.7.	

^1^ free amino nitrogen.

**Table 2:** The applied micromalting scheme of wheat samples.

Day	Phase	Malting Procedure		
		A	B	C
1st	Immersion steeping Dry steeping	5 h; t = 14.0 °C; 19 h; t = 14.5 °C		
2nd	Immersion steeping Dry steeping	4 h; t = 14.0 °C; 20 h; t = 14.5 °C		
3rd *	Immersion steeping	2 h; t = 14.0 °C;		
4th	Germination: relative humidity of air in each procedure: r.H. = 85%; sampling during germination was performed daily	t = 14.5 °C	14.5 °C	18.0 °C
5th			16.0 °C	17.0 °C
6th			17.0 °C	16.0 °C
7th			18.0 °C	14.5 °C
8th	Kilning: 19 h (after last hour of germination, kilning was employed and lasted for 19 h; malt was degerminated followed with packaging the samples into paper bags; stored for 2 months before the analysis			

* Control of the degree of steeping at the beginning of the third day and every hour of immersion steeping, when it was found that the grain does not tolerate any further soaking under water, the moisture content in malting procedure A, B, C of (A = 44.5%; B = 44.0%; C = 45.0%) was adjusted with sparging (spray steeping) in germination box (1st day of germination).

**Table 3:** Quality characteristics of the used wheat cultivars.

	Quality Indicator	Variety															
		1	2	3	4	5	6	7	8	9	10	11	12	13	14	15	16
1	Moisture (%)	12.03d	12.51b	11.92de	11.95de	11.71ef	11.68fg	11.69fg	12.9a	11.6gh	11.32i	11.83ef	10.73j	12.03de	12.3c	11.49hi	12.56b
2	1000 grain weight (g)	37.91k	43.98e	41.72g	38.57j	44.59d	35.17n	38.42j	43.12f	36.09m	48.82c	40.69h	49.66b	39.93i	36.8l	43.09f	57.79a
3	Total N (% dm)	1.92efg	1.7h	2.11bcd	1.82gh	1.76fgh	2.21b	2.05bcde	1.8h	2.22b	2.08bcde	2.4a	1.95defg	2.17bc	2.02cdef	1.92gh	2.12bcd
4	Total proteins (% dm)	10.94f	9.69i	12.03c	10.37g	10.03h	12.6b	11.69de	10.26	12.65b	11.7cd	13.68	11.12f	12.37b	11.51e	10.94f	12.08c
5	NIR-HD grain hardness	57gh	60fg	64de	56h	60fg	69ab	70a	62ef	65cde	68abc	65cde	70a	68abc	70a	66bcd	57gh
6	Total pentosanes (%dm)	8.01a	7.42bc	6.68f	6.82f	6.68f	6.91ef	7.24f	6.47f	7.41b	7.34bc	7.31bc	7.08de	7.41bc	7.35f	7.23cd	6.71f
7	Soluble pentosanes (%dm)	0.8abc	0.85a	0.81a	0.73bcde	0.72cde	0.72cde	0.73bcde	0.62fg	0.62efg	0.78abc	0.57g	0.69def	0.79abc	0.77abcd	0.81ab	0.66ef
8	Total/Soluble pent. (%)	10bcd	11.45a	12.1ab	10.65cde	10.77bcde	10.4defg	10.08bfgh	9.6h	8.36i	8.44i	7.8i	9.74gh	10.66cde	10.47cdef	11.2bc	9.84fgh
9	Vitreosity (%)	32cd	49a	19fg	20efg	5h	21ef	24e	19fg	16g	36c	32cd	51a	41b	30d	22ef	19fg

Values are the means of two measurements. Values displayed in the same lines and tagged with different letters are significantly different (*p* < 0.05).

**Table 4:** Mean values of the testing varieties for the quality parameters of the micromalting process by season and malting procedures.

		Malting Procedures			
		A (2018)	B (2018)	C (2018)	Recommended Values
1.	moisture after 48 h (%)	38.6a ± 050	37.61a ± 0.46	40.5a ± 0.35	>40%
2.	respiration losses (% g/dm)	1.76d ± 0.30	0.75c ± 0.35	2.87d ± 0.30	-
3.	germination losses (% g/dm)	4.80c ± 0.27	2.79b ± 0.7	4.49c ± 0.65	-
4.	total losses (% g/dm)	6.57b ± 0.24	3.54b ± 0.36	7.36b ± 0.28	<10%

Values are the means of two measurements ± standard deviation. Values displayed in the same lines and tagged with different letters (a–d) are significantly different (*p* < 0.05).

**Table 5:** Results of micromalting analysis (A procedure).

	Quality Indicator	Variety															
		1	2	3	4	5	6	7	8	9	10	11	12	13	14	15	16
1.	moisture after 48 h (%)	39.5d	37.6g	37.7g	40.7b	39e	40.6b	40.1c	37.4gh	40.4bc	39.6d	39.7d	37.5g	38.7ef	41.1a	38.5f	37.18h
2.	respiration losses (% g/dm)	1.64k	1.09m	1.85i	1.78k	2.59d	2.84c	2g	1.32l	0.97n	2.23f	2.19g	2.81c	2.53e	2.95b	3.26a	1.06m
3.	germination losses % g/dm)	4.31g	2.52h	4.25g	6.5a	4.46f	6.1b	6.43a	5.06e	6.45a	5.71c	6.09b	5.3d	5.36d	5.03e	5.33d	4.35fg
4.	total losses (% g/dm)	5.95k	3.61m	6.13j	3.61m	6.13j	8.94a	8.43c	6.38i	7.41h	7.94g	8.28d	8.11e	7.98f	7.98f	8.59b	5.41l

Values in Table 5, Table 6 and Table 7 are the means of two measurements. Values displayed in the same lines and tagged with different letters are significantly different (*p* < 0.05).

**Table 6:** Results of micromalting analysis (B procedure).

	Quality Indicator	Variety															
		1	2	3	4	5	6	7	8	9	10	11	12	13	14	15	16
1.	moisture after 48 h (%)	37.1hi	37.4fg	37.8de	37.6ef	37.2gh	37.9cd	37.7de	37.4fg	38.4b	37.4fg	38.1c	37.3gh	37.8de	38.9a	37.4fg	36.9i
2.	respiration losses (% g/dm)	0.76h	0.57i	1.11c	0.41j	0.94fg	1.03d	1.02de	0.91g	0.52i	0.05k	1.01de	1.32b	0.94fg	0.1k	1.7a	0.97ef
3.	germination losses % g/dm)	1.8h	2.17g	2.15g	3.26c	2.15g	3.59b	3.93a	3.25cd	3.58b	3.22de	3.23cd	3.19e	3.95a	2.9f	3.95a	1.8h
4.	total losses (% g/dm)	2.55o	2.73n	3.27j	4.66i	3.09k	4.63d	4.95b	4.16g	4.08h	3.26j	4.24f	4.51e	4.88c	3.00l	5.65a	2.77m

Values in Table 5, Table 6 and Table 7 are the means of two measurements. Values displayed in the same lines and tagged with different letters are significantly different (*p* < 0.05).

**Table 7:** Results of micromalting analysis (C procedure).

	Quality Indicator	Variety															
		1	2	3	4	5	6	7	8	9	10	11	12	13	14	15	16
1.	moisture after 48 h (%)	39.6g	39.8g	41.3abcd	41.4abc	40g	40.8ef	40.6f	39.1h	41.2bcde	41cdef	41.5ab	39.8g	40.6f	41.7a	40.9def	38.77h
2.	respiration losses (% g/dm)	2.24k	3.07e	3.91c	2.15l	2.64h	4.52a	2.51i	2.35j	1.82m	1.69n	3.01f	3.38d	2.99g	2.64h	4.11b	1.62o
3.	germination losses % g/dm)	3.24m	3.24m	4.29i	5.8c	3.94k	6.79a	5.37e	4.34h	6.45b	5.39e	5.72d	5.3f	5.03g	3.99j	5.72d	3.63l
4.	total losses (% g/dm)	5.48o	6.31n	8.2f	7.95h	6.58m	11.31a	7.88i	6.69k	8.28e	7.08j	8.73c	8.69d	8.02g	6.62l	9.83b	5.25p

Values in Table 5, Table 6 and Table 7 are the means of two measurements. Values displayed in the same lines and tagged with different letters are significantly different (*p* < 0.05).

**Table 8:** Mean values of the testing varieties for the quality parameters of the malt.

		Recommended Values	Malting Procedures		
			A	B	C
1.	1000 grain weight (g d.wt.)	-	35f ^1^ ± 0.25	35.1f ± 5.24	33.2f ± 5.06
2.	Vitreosity (%)	5–10 ^4^	6g ± 5.91	8g ± 6.74	3g ± 4.47
3.	Total N (% d.wt.)	>1.8 ^5^	1.67g ± 0.25	1.69g ± 0.19	1.8g ± 0.14
4.	Soluble N (mg/L)	700–900 ^2^	660a ± 47.92	582a ± 39.98	690a ± 53.44
5.	Kolbach Index (%)	<42 ^4^	40.0f ± 3.34	34.24f ± 4.16	37.9f ± 2.72
6.	FAN (mg/100 g dry wt.)	80–110 ^2^	125c ± 7.35	117c ± 6.33	132c ± 5.84
7.	Fine extract content (% d.wt.)	-	85.9d ± 1.65	85.47d ± 1.64	89.68d ± 1.9
8.	Extract difference (%)	<2.5 ^4^	1.6g ± 0.88	2.09g ± 1.02	1.1g ± 0.86
9.	Wort color (EBC u.)	3–5 ^3^	4.7g ± 0.96	4.4g ± 0.36	4.2g ± 0.68
10.	Filtration time (min)	<60 ^3^	58e ± 16.90	66e ± 19.36	49e ± 9.64
11.	pH	5.9–6.1	6.2g ± 0.06	6.3g ± 0.06	6.2g ± 0.09
12.	Viscosity (mPas. 8.6%e)	<1.8	1.563g ± 0.07	1.642g ± 0.07	1.537g ± 0.07
13.	Hartong number VZ 45 °C (%)	>33 ^3^	33f ± 4.41	28.1f ± 2.86	36.9f ± 4.33
14.	Diastatic power WK°	250–420	256b ± 8.42	236b ± 6.26	265b ± 7.51
15.	Final attenuation of wort (%)	≈78 ^3^	82.8d ± 0.73	80.4d ± 1.04	83.9d ± 0.94

^1^ Values are the means measurements ± standard deviation. Values displayed in the same lines and tagged with different letters (a–g) are significantly different (*p* < 0.05); ^2^ [12], ^3^ [15]; ^4^ [27]; ^5^ [28].

**Table 9:** Results of malt analysis (A procedure).

Quality Indicator	Variety															
	1	2	3	4	5	6	7	8	9	10	11	12	13	14	15	16
Moisture (%)	5.60a	5.1fg	4.95i	5.25e	5.28e	5.42c	5.09g	5.11fg	4.75j	5.27e	5.49b	5.35d	5.15f	5.24e	4.62k	5.01h
1000 grain weight (g d.wt.)	31.7m	38.2e	34.0j	38.6d	36.5g	29.2p	32.2l	34.8g	31.0n	42.6a	34.2i	39.4c	32.4k	29.4o	34.6h	40.4b
Vitreosity (%)	8cd	5e	8cd	4ef	2fg	6de	0g	4ef	0g	16b	22a	10c	2fg	2fg	0g	2fg
Total N (% d.wt.)	1.92c	1.31j	1.32j	1.55h	1.63fg	1.44i	1.62g	1.52h	1.73d	1.46i	1.64fg	1.92c	2.17a	1.67ef	1.70de	2.1b
Soluble N (mg/L)	638f	572.3h	564h	612g	646f	657e	657e	639f	706c	643f	690d	705c	687d	686d	717b	740a
Kolbach Index (%)	35.5i	43.5b	42.6c	39.5g	39.5g	45.6a	40.6f	42.1de	40.7f	40.7f	41.7e	40.9f	32.9j	36.3h	42.3cd	35.6i
FAN (mg/100 g dry wt.)	140.3a	130.1bcd	130.1bc	128.2cde	123.4efg	115.3hi	117.4gh	110.3i	118.4fgh	135.4ab	127.2cde	127.4cde	120.4fgh	124.1def	130.4ef	126.3efg
Fine extract content (% d.wt.)	82.6j	86.8bc	82.43j	85.72fgh	85.72fgh	88.34a	88.73a	86.48cde	87.23b	85.37gh	86.68bcd	86.14def	85.28h	85.89fg	86.1ef	84.31i
Extract difference (%)	2.92b	2.05d	3.15a	1.33f	2.98b	0.72i	1.03g	1.64e	0.83hi	0.83hi	0.32j	0.94gh	2.33c	2.13d	1.23f	1.75e
Saccharification time (min)	10–15	15–20	20–25	<10	15–20	15–20	15–20	<10	15–20	10–15	10–15	10–15	<10	10–15	10–15	<10
Wort colour (EBC u.)	8.9k	4.4ef	4.8d	4.3fg	4.0ij	5.0c	4.5e	3.9j3.6j	4.5e	8.6a	4.0ij	5.0c	4.1hi	4.2gh	8.0b	4.8d
Filtration time (min)	40.0g	65.0c	65.0c	55.0e	60.0d	70.0b	60.0d	65.0c	60.0d	105a	70.0b	30.0gh	50.0f	50.0f	50.0f	40.0g
pH	6.2ab	6.2ab	6.2ab	6.2ab	6.2ab	6.2ab	6.2ab	6.3a	6.3a	6.3a	6.3a	6.1b	6.3a	6.29a	6.2ab	6.3a
Viscosity (mPas. 8.6%e)	1.4993k	1.59f	1.667a	1.6247e	1.645c	1.484m	1.488l	1.634d	1.505j	1.662b	1.533i	1.589f	1.546h	1.576g	1.463n	1.508j
Hartong number VZ 45 °C (%)	30.82i	27.4m	26.1n	30.5j	25.74o	32.2h	37.25c	27.9l	41.5a	34.6f	29.6k	36.6d	34.2g	34.9e	35.0e	37.65b
Diastatic power (°WK)	251.3gh	253fgh	247h	259.6cde	251.6gh	236i	256efg	251gh	262bcde	268.0ab	253fgh	268.6a	265m	258def	2606cde	263abcd
Final attenuation of wort (%)	82.3e	82.6de	83.5b	84.1a	82.2e	82.6de	82.9cd	83.4b	83.5b	82.7cde	82.8cd	80.7f	82.3e	84.2a	82.6cde	83.1bc

Values are the means of two measurements. Values displayed in the same lines and tagged with different letters are significantly different (*p* < 0.05).

**Table 10:** Results of malt analysis (B procedure).

Quality Indicator	Variety															
	1	2	3	4	5	6	7	8	9	10	11	12	13	14	15	16
Moisture (%)	5.0cd	5.24b	4.99def	5.1c	5.0cd	5.26b	4.73g	4.89f	4.6h	5.0cde	5.18b	5.28b	5.39a	5.33ab	4.7gh	4.9ef
1000 grain weight (g d.wt.)	31.7h	41.4b	33.3g	30.0j	38.1d	29.6k	30.4j	35.2f	31.6h	40.6c	33.3g	40.8c	30.9i	30.0j	36.6e	48.4a
Vitreosity (%)	10bc	10bc	14b	2d	0.6d	10bc	0d	4cd	0d	24a	16b	14b	10bc	6cd	0d	2d
Total N (% d.wt.)	1.94b	1.62b	1.85b	1.58b	1.66b	1.54b	1.44b	1.5b	1.57b	1.5b	1.65b	1.92b	1.9b	1.7b	3.27a	2.06ab
Soluble N (mg/L)	659a	558efg	535fgh	587cd	603bc	589cd	573de	534gh	617b	556efgh	562def	650a	529h	602bc	534gh	621b
Kolbach Index (%)	34fgh	34.4fg	28.9j	37.3cd	36.4de	38.2bc	40.4a	35.5ef	39.3ab	37.1cd	34fgh	33.9gh	25.4k	31.8i	32.8hi	29.5j
FAN (mg/100 g dry wt.)	127.4a	123.5abcd	125.6ab	122.4abcde	117.3efg	108.7hi	109.6hi	106.8i	112.3ghi	124.1abc	119.9bcde	119.5cde	112.2ghi	112.2ghi	118.1def	113.5fgh
Fine extract content (% d.wt.)	84.45ghi	88.2a	83.5i	85.13efg	84.8fgh	87.9ab	87.8ab	85.8def	86.9bcd	85.4efg	87.2abc	86.1cde	84.4ghi	84.9fgh	83.9hi	81.86j
Extract difference (%)	2.9c	1.85f	4.3a	1.53g	2.18e	0.83j	1.02i	2.36d	1.95f	1.84f	1.94f	2.37d	4.0b	2.45d	0.59k	1.24h
Saccharification time (min)	10–15	15–20	20–25	10–15	10–15	20–25	15–20	20–25	15–20	25–30	25–30	10–15	20–25	20–25	20–25	20–25
Wort colour (EBC u.)	4.0f	4.3e	4.5cd	5.0a	4.5cd	3.7g	4.7b	4.1f	4.6bc	5.1a	4.3e	4.4de	4.5cd	4.4de	4.3e	4.1f
Filtration time (min)	40l	70f	60h	75e	60h	55i	50j	65g	60h	110a	80d	45k	90c	50j	95b	55i
pH	6.3b	6.3b	6.2c	6.2c	6.3b	6.3b	6.3b	6.3b	6.3b	6.3b	6.4a	6.2c	6.4a	6.3b	6.3b	6.3b
Viscosity (mPas. 8.6%e)	1.529k	1.556j	1.688cd	1.6217fg	1.569ij	1.597gh	1.556j	1.707bc	1.701c	1.787a	1.611gh	1.589hi	1.664de	1.69c	1.727b	1.643ef
Hartong number VZ 45 °C (%)	30.1bc	27.0def	22.6h	25.4fg	24.2fg	33.4a	24.2gh	31.2b	28.7cd	26.4ef	30.3bc	30.5b	30.2bc	27.5de	28.2d	30.4b
Diastatic power (°WK)	225.3g	231.3def	228.6fg	241ab	234de	226g	231ef	236cd	241ab	241ab	235.3cde	241ab	245a	239bc	241ab	242.3ab
Final attenuation of wort (%)	80.5abcd	80.5abcd	79.4bcde	78.4de	78.6cde	80.4abcd	80.6abc	81.2ab	81.6a	80.6abc	81.5ab	77.8e	80.3abcd	81.7a	80.3abcd	82.1a

Values are the means of two measurements. Values displayed in the same lines and tagged with different letters are significantly different (*p* < 0.05).

**Table 11:** Results of malt analysis (C procedure).

Quality indicator	Variety															
	1	2	3	4	5	6	7	8	9	10	11	12	13	14	15	16
Moisture (%)	4.57j	5.5b	5.45bc	5.6a	5.42c	5.38c	5.1fg	5.05g	4.69i	5.15ef	5.19de	5.22de	4.93h	5.04g	4.27k	5.25d
1000 grain weight (g d.wt.)	29.4i	37.5d	39.3c	37.5d	29.0i	28.0j	31.5g	33.4f	30.1h	38.0d	31.6g	40.0b	31.4g	30.3h	34.0e	43.5a
Vitreosity (%)	4.0bc	0.0d	2.0cd	4.0bc	2.0cd	4.0bc	0.0d	0.0d	0.0d	14.0a	14.0a	6.0b	2.0cd	0.0d	0.0d	0.0d
Total N (% d.wt.)	1.96ab	1.89abc	1.61de	1.96ab	1.46e	1.81bc	1.72cd	1.77bcd	1.79bcd	1.81bc	1.72cd	1.85bc	1.87abc	1.7cd	1.87abc	2.05a
Soluble N (mg/L)	694.0d	774.0a	622.0g	719.0c	645.0f	723.0c	692.0d	598.0h	742.0b	622.0g	617.0g	765.0a	723.0c	676.0e	699.0d	734.0bc
Kolbach Index (%)	35.4gh	41.0abc	38.7de	36.7fg	40.1bcd	39.9cd	41.8a	33.8i	41.6ab	34.4hi	38.0ef	40.8abc	35.4gh	35.3ghi	37.5gh	35.8gh
FAN (mg/100 g dry wt.)	145.5a	136.6c	136.5c	140.1b	135.5c	121.5h	128.6fg	130.2ef	127.55fg	128.2fg	134.2cd	136.1c	126.5g	131.7de	134.2cd	128.2fg
Fine extract content (% d.wt.)	84.4h	86.6def	92.4a	85.7fgh	86.5def	88.4bc	88.7b	86.7de	87.0cde	86.1defg	87.3bcd	86.3def	84.7gh	85.2fgh	85.7efgh	84.7gh
Extract difference (%)	1.4cd	3.5a	1.2de	1.27de	1.0ef	0.55g	0.1h	1.1de	0.2h	0.41gh	0.62g	0.62g	1.95b	1.7bc	0.72fg	1.23de
Saccharification time (min)	<10	<10	10-15	<10	<10	<10	<10	<10	<10	<10	<10	<10	<10	<10	<10	<10
Wort colour (EBC u.)	3.4h	3.4h	3.3h	3.8g	4.6cd	4.5d	4.0f	4.0f	4.0f	5.8a	3.4h	5.1b	4.3e	4.3e	4.0e	4.7c
Filtration time (min)	45e	50d	70a	40f	40f	50d	60b	60b	50d	60b	55c	40f	40f	45e	35g	50d
pH	6.3a	6.3a	6.3a	6.0d	6.1c	6.2b	6.1c	6.2b	6.1c	6.2b	6.2b	6.1c	6.2b	6.2b	6.1c	6.1c
Viscosity (mPas. 8.6%e)	1.582b	1.549cd	1.483f	1.560cd	1.5437d	1.456g	1.455g	1.584b	1.461g	1.596b	1.514e	1.697a	1.561c	1.586b	1.446g	1.514e
Hartong number VZ 45 °C (%)	39.5bc	36.0de	30.1h	32.3fg	36.8d	33.5f	40.6b	32.7fg	40.7b	38.5c	31.9g	44.45a	33.2f	39.5bc	35.3e	44.4a
Diastatic power (°WK)	257a	263a	192.3b	267a	262a	245.3a	265a	261a	270a	275a	267a	275.6a	275a	265a	270a	269a
Final attenuation of wort (%)	84.0b	83.6b	84.1b	84.7b	83.5b	83.6b	83.7b	84.1b	84.8b	83.7b	83.9b	82.212	83.6b	85.2b	82.8	83.6

Values are the means of two measurements. Values displayed in the same lines and tagged with different letters are significantly different (*p* < 0.05).

## Data Availability

Restrictions apply to the availability of these data. Data was obtained from the Agricultural Institute Osijek and are available with the permission of the Agricultural Institute Osijek.
